# A Lymphocyte Subset-Based Prediction Model for Refractory Community-Acquired Pneumonia in Immunocompetent Patients

**DOI:** 10.3390/diagnostics15131627

**Published:** 2025-06-26

**Authors:** Jingyuan Zhang, Xinyu Hu, Ailifeila Aili, Lei Pan, Xinying Xue, Xiaolan Chen

**Affiliations:** Department of Respiratory and Critical Care Medicine, Emergency and Critical Care Medical Center, Beijing Shijitan Hospital, Capital Medical University, Beijing 100038, China; ldartemis@outlook.com (J.Z.); bjhuxinyu@126.com (X.H.); elvis_vira@163.com (A.A.); leipan61@aliyun.com (L.P.); xuexinying2988@bjsjth.cn (X.X.)

**Keywords:** refractory community acquired pneumonia, T lymphocyte, CD4^+^ T cell, CD8^+^ T cell, double negative T cell

## Abstract

**Background/Objectives**: Refractory community-acquired pneumonia (r-CAP) has become a thorny issue in clinical practice, especially after the COVID-19 pandemic, even in immunocompetent patients, as conventionally defined. In this study, we aimed to identify the risk factors for immunocompetent patients with r-CAP. **Methods:** This was a single-center retrospective study. In total, we collected clinical data from 82 patients with r-CAP in whom the first-line antibiotic therapy failed and 82 patients with general CAP (g-CAP) who recovered with first-line antibiotics, matched at a ratio of 1:1, admitted to Beijing Shijitan Hospital, Capital Medical University, from 1 January 2022, to 31 December 2023. The differences between the two groups (clinical characteristics, peripheral blood cell count, lymphocyte subsets, and regular laboratory indicators) were analyzed using paired *t*, paired Wilcoxon, Chi-square, or Fisher’s exact tests, and univariate and multivariate logistics regression analyses were conducted to identify the independent risk factors. A model for predicting indicators with statistical significance was established and proved with the receiver operating characteristic (ROC) curve. **Results**: Warm season, a history of chronic obstructive pulmonary disease, longer time from onset to admission (T_O-A_), higher percentages of CD4^+^ T, CD8^+^ T, and double-negative T (DNT) lymphocytes, as well as higher levels of C-reactive protein (CRP), low-density lipoprotein cholesterin (LDL-C), serum sodium ion (Na^+^), and free-calcium ion (FCa^2+^) were regarded as independent risk factors, while T lymphocyte percentage (T%) and total cholesterol (TC) were identified as protective factors. The combined multivariate model using all the above factors proved to be sensitive and specific (AUC = 0.8711, *p* < 0.0001, R^2^ = 0.4235), and thus better than the respective univariate models. **Conclusions**: Increased CD4^+^ T%Lym, CD8^+^ T%Lym, and DNT%Lym, warm season, a history of COPD, longer T_O-A_, and increased levers of CRP, LDL-C, Na^+^, and FCa^2+^ potentially cause CAP to be refractory, while the T lymphocyte count, namely, the overall cellular immunity, was impaired in r-CAP patients, and increased TC levels could be beneficial to pneumonia recovery.

## 1. Introduction

Community-acquired pneumonia (CAP), as the third-leading cause of death and the leading cause of infections, brings about approximately three million deaths every year across the world [1]. The first-choice antibiotics for CAP include quinolones, semisynthetic penicillin, tetracyclines, etc., with local microbiological epidemiology considered [2]. Refractory community-acquired pneumonia (r-CAP) is defined as CAP wherein the first-choice antibiotics failed, leading to admission to senior hospitals or even intensive care units, which causes heavy burdens to families and society.

There is no consensus regarding the definition of immunosuppression in community-acquired pneumonia [3]. A global initiative has defined immunocompromised patients with CAP as patients with at least one of the following risk factors: acquired immune deficiency syndrom (AIDS), aplastic anemia, asplenia, hematological cancer, chemotherapy during the past 3 months, neutropenia, biological drug use, lung transplantation, chronic steroid use (>10 mg/day of prednisone or ≥3 months before hospital admission), lung cancer with either neutropenia or chemotherapy, and another solid tumor with either neutropenia or chemotherapy [4]. The spectrum of potential pathogens causing CA may be expanded according to the type and severity of immunosuppression to include fungal infections, less common viral infections, and even parasitic infections [4]. Thus, r-CAP is not rare in conventionally defined immunosuppressed patients, so accurate causal examinations [e.g., next-generation sequencing (NGS) of sputum, bronchoalveolar lavage fluid (BALF), or blood] are usually conducted in advance to avoid deteriorated conditions or even death and antibiotic misuse or abuse. However, during clinical practice, we observed that increasing conventionally defined immunocompetent patients showed no response to first-choice antimicrobial medicines and that further pathogen examinations proved their infections to be from uncommon opportunistic or multi-drug-resistant pathogens, especially after the COVID-19 pandemic.

Lymphocytes consist of the immune system including B cells, which mediate the humoral immunity by secreting pathogen-neutralizing antibodies and proinflammatory cytokines (plasm cells) [5] and providing long-lasting protection (memory B cells) [6]; T cells, which induce cellular immunity by inducing virus-infected cell death (CD8^+^ T cells) [7,8] and modifying the activity of other immune cells to clear infection (CD4^+^ T cells) [9,10,11,12]; and natural killer (NK) cells, which participate in innate immune responses, especially to viral infections [13]. Although more attention has recently been paid to the number and proportion of different lymphocyte subsets in immunocompromised patients, no research has elucidated how the immune conditions in so-called immunocompetent patients change during infection and why CAP becomes refractory.

In this study, we analyzed and compared the clinical characteristics and laboratory indicators, including lymphocyte subsets, in immunocompetent patients with general CAP (g-CAP) and r-CAP so as to help clinical practitioners identify possible r-CAP patients in advance and to show the need for further precise microbiological tests (e.g., next-generation sequencing) for earlier precise treatments to alleviate patient sufferings, shorten hospital stays, and reduce total costs.

## 2. Materials and Methods

This is a single-center, retrospective, observational study. From 1 January 2022, to 31 December 2023, we included patients with CAP admitted to Beijing Shijitan Hospital, Capital Medical University.

The inclusion criteria were (1) a diagnosis of CAP; (2) age ≥16 y/o; (3) complete data, including demographic data, complete blood cell count, laboratory indicators, and lymphocyte subsets.

The exclusion criteria were (1) malignant tumors (solid tumor or hematological cancer), (2) infections at other sites, (3) primary/acquired immunodeficiency diseases (e.g., AIDS), (4) treatment with immunosuppressive agents [such as disease-modifying anti-rheumatic drugs (DMARDs), biologics, chemotherapy, etc.] within the last 3 months, (5) treatment with chronic steroids (>10 mg/day of prednisone or ≥3 months before hospital admission), (6) lymphocyte infusion or hematopoietic stem cell transplantation in the last 3 months, (7) other hematological diseases (such as aplastic anemia, neutropenia, monoclonal gammopathy of undetermined significance, etc.), or (8) end-of-life care [3,4].

The patients were divided into g-CAP and r-CAP groups. The diagnostic criteria for CAP and severe CAP (SCAP) were based on the CAP guidelines of the Chinese Medical Doctor Association [14], Chinese Thoracic Society [15], and National Institute for Health and Care Excellence [2], and r-CAP patients were defined accordingly as those who showed no response to the first-choice antimicrobial medicines, including quinolones, semisynthetic penicillin, tetracyclines, macrolides, and anti-viral medicines according to local seasonal epidemiology (e.g., neuraminidase inhibitors, Nematovir/Litonavir, Molnupiravir, Azvudine, Simnotrelvir/Ritonavir, Atilotrelvir/Ritonavir, etc.), combined or not combined with first- or second-generation cephalosporins. No response to any of the above medicines was defined as unimproved fever, sputum, wheezing, or hemoptysis for more than 3 days; a decrease in CRP of less than 30% in 3 days; or unabsorbed or enlarged lung lesions (e.g., exudation, consolidation, ground-glass opacity, etc.) for more than 1 month, with all the above improved with adjusted antimicrobial regimens based on microbiological and drug susceptibility tests. The patients were matched at a ratio of 1:1, using the Charlson Comorbidity Index (CCI) based on myocardial infarction, congestive heart failure, peripheral vascular disease, cerebrovascular accident or transient ischemic attack, dementia, chronic pulmonary disease, connective tissue disease, peptic ulcer disease, liver disease, diabetes with or without end-organ damage, hemiplegia, and chronic kidney disease [16].

Following data were collected.

(1) Demographic data: We collected clinical data, including age, gender, onset season, antimicrobial regimen before admission, past history [e.g., chronic obstructive pulmonary disease (COPD), diabetes mellitus, hypertension, hyperlipemia, COVID-19 infection history (in the past 3 months), smoking history, etc.] from electronic medical records. The severity of pneumonia was evaluated by CURB-65 scores based on consciousness, blood urea nitrogen, respiratory rate, blood pressure, and age, as well as pneumonia severity index (PSI) according to age, mental status, pulse, respiratory rate, systolic blood pressure <90 mmHg, temperature, history, demographics, comorbidity, physical exam findings, and lab and radiographic findings.
(2) For each patient with CAP, the onset date was divided into cold or warm seasons based on the climate characteristics of Beijing, that is, the 6 months with the highest monthly average temperatures were considered the warm season, and other months were considered the cold season.
(3) Laboratory indicators: The laboratory indicators included CBC count by a hematology analyzer (BC-7500; Mindray), biochemical indicators [including TC, total triglyceride (TG), LDL-C, high-density lipoprotein cholesterin (HDL-C), serum Na^+^, serum potassium (K^+^), serum chloride (Cl^−^), uric acid (UA), serum iron (Fe), total serum calcium (TCa^2+^), calculated serum calcium (CCa^2+^), FCa^2+^, and serum magnesium (Mg^2+^)] and CRP by Clinical Chemistry Analyzers (AU5832; Beckman Coulter), and lymphocyte subsets by flow cytometry (CytoFLEX S; Beckman Coulter), including T (CD3^+^), CD4^+^ T (CD3^+^CD4^+^CD8^−^), CD8^+^ T (CD3^+^CD4^−^CD8^+^), double-negative T (DNT) (CD3^+^CD4^−^CD8^−^), B (CD3^−^CD19^+^), and natural killer (NK) (CD3^−^CD56^+^) cells.
(4) Microbiological results: The microbiological data were collected from the sputum culture, BALF culture, and BALF NGS, etc.
(5) Statistic process: The percentage of lymphocytes in WBCs were calculated and represented as Lym%WBC. The percentages of respective lymphocyte subsets in all lymphocytes were calculated and represented as B%Lym, T%Lym, NK%Lym, CD4^+^%Lym, CD8^+^%Lym, and DNT%Lym. The pathogen detection rate was calculated as follows: detected pathogen number/total subject number in each group.

Ethics Approval: All patient information was confidentially protected, and this study was approved by the Ethics Committee of Beijing Shijitan Hospital, Capital Medical University. Informed consent forms were waived because this study is observational and retrospective.

Statistical Analysis: All data were analyzed with GraphPad Prism 10.0 (Graphpad, CA). Quantitative data are expressed as mean ± standard deviation, and differences were compared with a paired t test. Nonparametric variables are expressed as medians and interquartile ranges, and a paired Wilcoxon test was used for significance. Continuous and categorical variables are summarized as counts and percentages, and Chi-square or Fisher’s exact tests were used for comparison between groups. Univariate and multivariate logistic regression models were used to identify possible factors associated with r-CAP. Receiver operating characteristic (ROC) curves were used to show the sensitivity and specificity of factors with significance, separately and collectively. A (two-sided) *p* value <0.05 was considered statistically significant.

## 3. Results

### 3.1. Demography

A total of 164 patients were included and matched based on the inclusion, exclusion, and matching criteria mentioned above, with 82 patients each in the r-CAP and g-CAP groups. The demographic data are summarized and analyzed in Table 1. Compared with g-CAP, r-CAP tended to occur in warmer seasons (50.00% vs. 32.93; *p* = 0.0265) and COPD patients (21.95% vs. 8.54%; *p* = 0.0169). Patients with r-CAP generally experienced longer time from onset to admission and final diagnosis, differing among individuals (r-CAP: 49.85 ± 107.6, g-CAP: 11.48 ± 15.77; *p* < 0.0001). Additionally, more antimicrobial regimens were prescribed to r-CAP patients before ultimate accurate pathogen tests, especially with first- and second-generation cephalosporins (28.05% vs. 3.66%; *p* < 0.0001) and carbapenem (10.98% vs. 0.00%; *p* = 0.0020). However, more patients in the g-CAP group were cured with anti-SARS-CoV-2 medicines (7.32% vs. 0.00%; *p* = 0.0126), which should be attributed to the prevalence and accuracy of SARS-CoV-2 tests.

No significant differences were found in gender (*p* = 0.6337), age (g-CAP: 60.77 ± 18.70; r-CAP: 61.15 ± 17.84; *p* = 0.5398), smoking history (25.61% vs. 36.59%; *p* = 0.1290), diabetes mellitus (20.73% vs. 23.17%; *p* = 0.7059), hypertension (40.24% vs. 41.46%; *p* = 0.8738), hyperlipemia (34.15% vs. 29.27%; *p* = 0.5021), or COVID-19 infection history in the past 3 months (12.20% vs. 6.10%; *p* = 0.1756) between the g-CAP and r-CAP groups.

Furthermore, for the severity of pneumonia at admission, more SCAP patients were found in the r-CAP group (18.29% vs. 0.00%; *p* < 0.0001), with increased possibility for deterioration (12.20% vs. 0.00%; *p* = 0.0011) than those with g-CAP. However, according to the classical prediction models, CURB-65 (*p* = 0.9911) and PSI (*p* = 0.1266), no statistically significant difference was found between the two groups.

### 3.2. Peripheral Lymphocyte Subsets

As for lymphocyte subsets, which reflect individual immune conditions, based on paired *t* or Wilcoxon tests, differences were discovered in CD4^+^ T lymphocyte count [g-CAP: 457.00 (298.30, 669.50), r-CAP: 546.50 (359.30, 751.50); *p* = 0.0397], CD4^+^ T cell percentage (g-CAP: 41.50 ± 8.19, r-CAP: 47.50 ± 8.53; *p* < 0.0001), and CD4^+^/CD8^+^ [g-CAP: 1.75 (1.30, 2.53), r-CAP: 2.20 (1.70, 3.03); *p* = 0.0033] between the two groups (Table 2).

### 3.3. Laboratory Indicators

To further explore the relative risk factors for r-CAP, we analyzed the clinical biochemical indicators between the two populations. In Table 3, the results show that serum lipid levels decreased in the r-CAP group, including TC [g-CAP: 4.08 (3.62, 4.71), r-CAP: 4.05 (3.16, 4.74); *p* = 0.0428] and TG [g-CAP: 1.25 (0.97, 1.95), r-CAP: 1.16 (0.79, 1.60); *p* = 0.0056]. When comparing serum ions, we found only serum Na^+^ [g-CAP: 138.00 (135.80, 140.00), r-CAP: 139.00 (136.00, 141.00); *p* = 0.0217] and free Ca^2+^ (FCa^2+^) [g-CAP: 1.08 (1.05, 1.11), r-CAP: 1.10 (1.06, 1.13); *p* = 0.0337] concentrations increased in r-CAP patients. The level of C-reactive protein (CRP), an indicator reflecting inflammatory conditions, significantly rose in the r-CAP group [g-CAP: 18.03 (4.85, 59.31), r-CAP: 41.06 (5.39, 106.2); *p* = 0.0334].

### 3.4. Prediction Models for Refractory Community-Acquired Pneumonia

To further explore the potential independent risk factors, we conducted univariate and multivariate logistic regression analyses. According to the univariate logistics regression analysis shown in Table 4, onset season (*p* = 0.0274), COPD history (*p* = 0.0207), time from onset to admission (*p* = 0.0029), CD4^+^ T lymphocyte count (*p* = 0.0288), CD4^+^ T lymphocyte percentage (*p* < 0.0001), CD4^+^/CD8^+^ ratio (*p* = 0.0142), Na^+^ (*p* = 0.0231), FCa^2+^ (*p* = 0.0206), and CRP (*p* = 0.0250) were regarded as potential risk factors for r-CAP, while TG (*p* = 0.0188) was screened out to inhibit the development of r-CAP. Furthermore, a multivariate logistic regression analysis proved that the risk factors for r-CAP included warm season (OR 5.341, *p* = 0.0281); a history of COPD (OR 62.280, *p* = 0.0019); longer time from onset to admission (OR 1.037, *p* = 0.0354); and increased percentages of lymphocyte (OR 1.525, *p* = 0.0122), CD4^+^ T lymphocyte (OR 3.044, *p* = 0.0003), CD8^+^ T lymphocyte (OR 2.621, *p* = 0.0006), and DNT lymphocyte (OR 2.188, *p* = 0.0420) and levels of LDL-C (OR 178.50, *p* = 0.0131), Na^+^ (OR 1.429, *p* = 0.0206), FCa^2+^ (OR 1,917,152, *p* = 0.0170), and CRP (OR 1.022, *p* = 0.0230), while increased T lymphocyte percentages (OR 0.386, *p* = 0.0012) and TC levels (OR 0.0086, *p* = 0.0173) could be protective factors against r-CAP.

**Figure 1:**
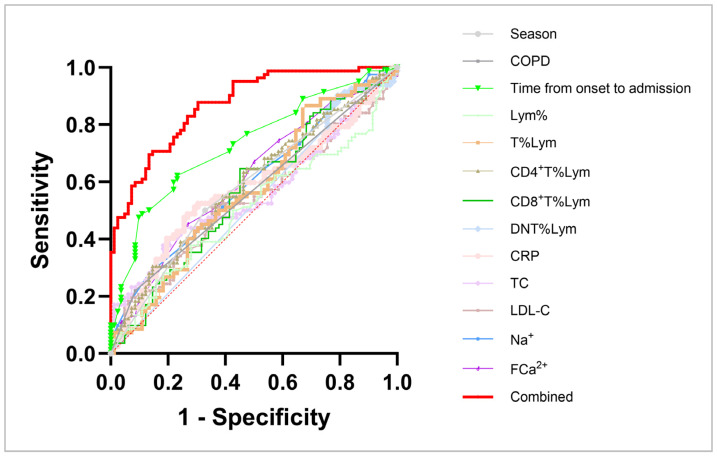
Receiver operating characteristic (ROC) curves of respective and combined independent factors in immunocompetent patients with r-CAP. COPD: chronic obstructive pulmonary disease; Lym: lymphocyte; DNT: double-negative T cell; TC: total cholesterol; LDL-C: low-density lipoprotein cholesterin; Na^+^: sodium; FCa^2+^: free calcium; CRP: C-reactive protein. The indicators of the combined curve include season; COPD history; time from onset to admission; percentages of T, CD4^+^ T, CD8^+^ T, and DNT cells; and levels of CRP, TC, LDL-C, Na^+^, and FCa^2+^.

Moreover, ROC curves were used to detect the sensitivity and specificity of the various mentioned independent correlative factors, with moderate AUC values (Table 5; Figure 1). To enhance the diagnostic power, a combined ROC curve comprising all correlative factors with statistical significance (season; COPD history; T_O-A_; percentages of CD4^+^ T, CD8^+^ T, and DNT cells; and levels of CRP; LDL-C; Na^+^; and FCa^2+^, as well as T lymphocyte percentage and TC level) was designed, with a remarkably improved prediction power (sensitivity = 75.61%, specificity = 76.83%; AUC = 0.8711, *p* < 0.0001; R^2^ = 0.4235) than those of the respective factors (Table 5 and Figure 1).

### 3.5. Microbiological Examination

**Figure 2:**
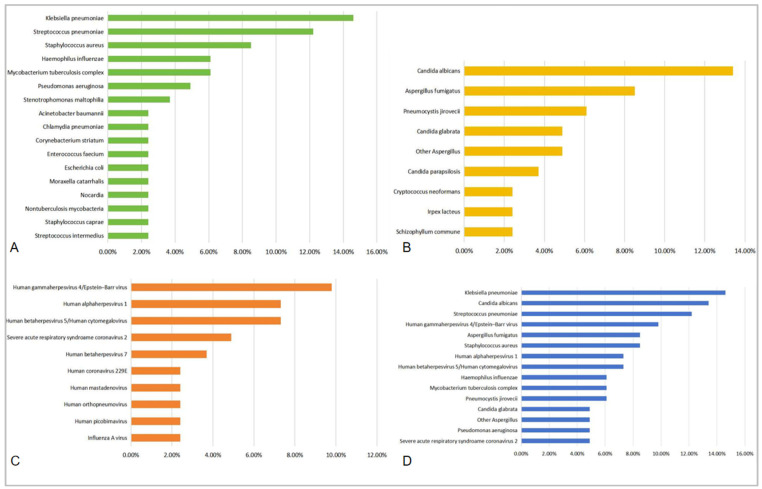
Pathogen rankings for r-CAP. (**A**) Bacterium ranking. (**B**) Fungus ranking. (**C**) Virus ranking. (**D**) Overall pathogen ranking.

To determine the reason for the failure of first-line antimicrobial therapies, we collected microbiological information from cultures (sputum or BALF) and NGS results. Th top 10 pathogens with detection rates above 1.2% (i.e., detection number > 1) were ranked, shown in Table 6 and Table 7. In Table 6 and Figure 2A, the most prevalent bacteria were found to be *Klebsiella pneumoniae* (14.6%), *Streptococcus pneumoniae* (12.2%), *Staphylococcus aureus* [8.5%, including *methicillin-resistant Staphylococcus aureus* (MRSA)], *Haemophilus influenzae* (6.1%), and *Mycobacterium tuberculosis complex* (6.1%). The highest detection rates for the fungi were found for *Candida albicans* (13.4%), *Aspergillus fumigatus* (8.5%), and *Pneumocystis jirovecii* (6.1%) (Table 6 and Figure 2B). Viruses were also not rare, but most of them were regarded as nonpathogenic, such as *Human gammaherpesvirus 4*/*Epstein-Barr virus* (*EBV*) (9.8%), *Human alphaherpesvirus 1*/*Herpes simplex virus type 1 (HSV-1)* (7.3%), and *Human betaherpesvirus 5*/*Human cytomegalovirus (CMV)* (7.3%) (Table 7 and Figure 2C). On the whole, bacteria (52.5%) were found to be most prevalent in r-CAP patients, followed by fungi (37.8%) and viruses (28.3%) (Table 7 and Figure 2D).

## 4. Discussion

Refractory community-acquired pneumonia is usually studied in traditionally defined immunocompromised patients, such as those with human immunodeficiency virus (HIV) infection; solid and hematological malignancies; long-term use of immunosuppressives, chemotherapy, radiotherapy, or organ transplantation; etc. [3]. In order to identify patients of competent immunity who may develop refractory community-acquired pneumonia that is resistant to first-choice antimicrobial regimens, we designed this study, wherein clinical characteristics such as onset in warmer seasons, a history of COPD, and a longer T_O-A_, as well as changes in T lymphocyte subsets, inflammatory indicators, CRP, and metabolic indicators, i.e., LDL-C, Na^+,^ and FCa^2+^, were regarded as risk factors for r-CAP. Moreover, a decreased T lymphocyte count was found to play a critical role, while no significant difference was discovered in B and NK lymphocytes. In addition, patients with higher TC levels were proved less likely to develop r-CAP.

The main reasons for developing r-CAP could be ascribed to newly prevalent pathogens (such as Gram-negative bacteria, fungi, or viruses) and antimicrobial resistance (AMR). The Institute for Health Metrics and Evaluation (IHME) pathogen core group has estimated that 704 million disability-adjusted life-years (DALYs) was correlated with 85 pathogens in 2019, globally, regionally, and for 204 countries and territories. The results showed that such a burden was responsible for 27.7% of the total DALYs from all causes in 2019 [17]. The leading pathogens, accounting for more than 50 million DALYs, included tuberculosis, malaria, and HIV or AIDS, while 18.5 million DALYs were attributed to fungi. Moreover, tuberculosis, *Streptococcus pneumoniae*, and *Staphylococcus aureus* were the top three bacteria, and some previously less recognized pathogens were observed to have considerable burden: *Staphylococcus aureus*, *Klebsiella pneumoniae*, *Escherichia coli*, *Pseudomonas aeruginosa*, *Acinetobacter baumannii*, and *Helicobacter pylori* [17]. These results are consistent with our findings, and less identified bacteria such as *Stenotrophomonas maltophilia*, *Chlamydia pneumoniae*, *Moraxella catarrhalis*, *Staphylococcus caprae*, *Enterococcus faecium*, *Corynebacterium striatum*, *Streptococcus intermedius*, *Nocardia*, and *Nontuberculosis mycobacteria* were observed to have high detection rates in r-CAP patients, most of which were recognized via NGS. Furthermore, the Global Burden of Diseases (GBD) Antimicrobial Resistance Collaborators has determined 4.71 million deaths to be associated with bacterial AMR (22 pathogens), with bacterial AMR being responsible for 1.14 million deaths from 1990 to 2021 [18]. The variations in AMR deaths were different by age and location, where for adults 70 years and older, mortality attributable to AMR was found to increase by more than 80%. Researchers have also predicted that approximately 1.91 million deaths ascribed to AMR and 8.22 million deaths correlated with AMR would occur globally in 2050, with an increase of 69.6% from 2022 to 2050. The greatest increase was discovered in MRSA for deaths correlated with and attributable to AMR, while among Gram-negative bacteria, resistance to carbapenems grew the most from 1990 to 2021 [18], results similar to those in our study.

COPD is commonly associated with persistent inflammation and injury of respiratory tracts. Molyneaux et al. have found an increase in bacterial burden and a considerable outgrowth of *Haemophilus influenzae* in patients with COPD after infection, compared with healthy subjects [19]. Other commonly isolated bacteria include *Moraxella catarrhalis*, *Streptococcus pneumoniae*, *Haemophilus parainfluenzae*, *Chlamydia pneumoniae*, and Gram-negative bacilli (i.e., *Pseudomonas aeruginosa*, *Escherichia coli*, *Proteus mirabilis*, etc.) [20,21,22,23,24,25]. On the other hand, adaptive immune responses participate in the development of COPD, where CD8^+^ cytotoxic T cells, in both the airways and alveolar compartments, are predominant, inducing structural cell death via apoptosis and necrosis, accompanied substantially by higher stages of airflow limitation and emphysema [26,27,28]. Furthermore, CD4^+^ T cells are accumulated in the airways and lungs of smokers with COPD, mainly including T helper (Th) 1 and Th17 cells, recruiting inflammatory cells to the lungs, associated with the severity of COPD [26,29,30,31].

Past research has reported that cytotoxic CD8^+^ T cells can be retained in airway mucosa, driven by an increase in T_EM_ (effectory memory T) cells and impaired apoptosis of T_RM_ (tissue-resident effectory memory T) cells, inducing lung injury, inflammation, airway remodeling, and other pathogeneses potentially correlated with complicated and severe pneumonia [9,32,33,34]. Such retention in both peripheral blood and parenchyma could be promoted by COPD, and viral infections (such as HIV, *influenza virus*, CMV, and SARS-CoV-2) [34,35,36,37,38]. In our study, viruses, especially human herpes virus (HHV), such as CMV, EBV, and HSV-1, were not rare but mostly recognized as nonpathogenic microbes. However, we also observed that when coinfected with such viruses, patients need an extended period of antimicrobial treatment, where CD8^+^ T cells may play an important role. Moreover, more studies are needed to determine the interaction between viruses and other pathogens in pneumonia. CD8^+^ T cells dynamically change in amounts and types at different stages of infection: (1) the expansion phase (0–7 days) with CD8^+^ T cells actively proliferating, (2) the peak of expansion (day 8) with effector CD8^+^ T cells up to the maximum, (3) the contraction phase (8–15 days) with majority of effector CD8^+^ T cells confronting apoptosis, and (4) the memory phase (>30 days) with only a small population of cells surviving and differentiated into various types of memory cells, namely, CD44^+^CD62L^−^ T_EM_, CD44^+^CD62L^+^ T_CM_, and CD69^+^CD103^+^ T_RM_ [7,39]. In the two studied groups, the average time from onset to admission was both longer than 11 days and no significant difference was found in the number of patients with T_O-A_ > 7 days (g-CAP vs. r-CAP: 64.63% vs. 78.05%), suggesting that the CD8^+^ T cells of most patients reached a peak. Furthermore, the percentage of CD8^+^ T cells of r-CAP patients was significantly increased compared with those of g-CAP patients, although more r-CAP patients experienced the memory phase of CD8^+^ T cells, where, theoretically, the CD8^+^ T cell count decreased to the trough. However, more studies are needed to identify specific CD8^+^ T cell subclusters to determine whether the absolutely enhanced CD8^+^ T cell activity or the prolonged expansion phase mainly participates in such a phenomenon.

During infection, naïve CD4^+^ T cells develop into distinct CD4^+^ Th cells, mainly including Th1, Th2, Th17, Th22, Tfh, and CD4^+^ cytotoxic T lymphocytes (CTLs), participating in pathogen clearance, by producing massive pro-inflammatory cytokines and chemokines [40,41]; exerting direct cytolysis [42,43]; and augmenting the functions of macrophages, CD8^+^ CTLs, B cell, and antibodies [7,42]. Their co-stimulation is commonly observed in various microbial infections, including viruses (e.g., CMV [44], SARS-CoV-2 [45,46], EBV [47], *Middle East respiratory syndrome coronavirus* (MERS-CoV) [48] and *influenza virus* [49,50]), fungi (e.g., *Aspergillus fumigatus* [51]), parasites (e.g., *Echinococcus multilocularis* [52]), and bacteria (e.g., *Mycobacterium tuberculosis* [53,54], and *Staphylococcus aureus* [55]), corresponding to our findings, but the relationship between T cells and pathogens needs to be explored further.

Double-negative T (DNT) cells (CD3^+^CD4^−^CD8^−^) can express either TCRαβ or TCRγδ and do not express NKT cell markers (such as CD16, CD56, CD49b, etc.), occupying approximately 3–5% of T lymphocytes in peripheral blood [56,57,58]. TCRαβ^+^ and TCRγδ^+^ T cells are usually antagonistic and modulate inflammation and immune responses via IL-10, IL-17, IFN-γ, and other cytokines during infection with *Leishmania* (skin), *Francisella tularensis* (lung), *Mycobacterium tuberculosis*, *simian immunodeficiency virus* (SIV), and HIV [57,58,59,60,61,62]. DNT cells were not commonly identified in our study, but the difference was found to be statistically significant between the two pneumonia subpopulations, possibly related to opportunistic pathogens, such as *Mycobacterium tuberculosis*.

Furthermore, Reijnders et al. have identified that peripheral monocytes are correlated with CAP and COVID-19 [63]. However, no significant difference was found in monocyte count and percentage in our study. This discrepancy might be attributed to the different onset periods where monocytes might take part in acute CAP at the initial stage, while lymphocytes may function later and persistently play a main role in chronic infection.

No prior study has explored the influential factors of r-CAP. Our study has proved that CAP becomes refractory in immunocompetent patients, especially with COPD, when their overall cellular immunity is impaired, while their CD4^+^ T, CD8^+^ T, and DNT cell percentages increase, indicating a possible co-infections with viruses, fungi, and opportunistic bacteria. Additionally, the prediction model has been verified to be effective, with favorable sensitivity and specificity. Therefore, in clinical practice, more precise tests on pathogens and drug susceptibility need to be conducted in patients with the r-CAP characteristics mentioned above to ensure suitable antimicrobial regimens are started earlier in order to alleviate individual pains and economic burdens. More explorations are needed to create a prediction model with lymphocyte subsets for specific pathogens in r-CAP.

However, some limitations remain in this study. First, the sample size was small, limited to a single center. Second, this was a retrospective study, so some subjects were excluded due to incomplete data, especially some with mild situations who had not been examined for lymphocyte subsets as a regular test. In order to solve this problem, we have matched the subjects according to their CCI scores, so as to reduce the influence of basic conditions as much as possible. Still, a prospective study is needed in the future to confirm the correlation and further explorations mentioned above.

## 5. Conclusions

Increased CD4^+^ T%Lym, CD8^+^ T%Lym, DNT%Lym, warm season, a history of COPD, longer T_O-A_, and increased levels of CRP, LDL-C, Na^+^, and FCa^2+^ potentially cause CAP to be refractory, while the T lymphocyte count, namely, the overall cellular immunity, were impaired in r-CAP patients, and increased TC levels could be beneficial to pneumonia recovery.

## Figures and Tables

**Table 1:** Demographic and clinical characteristics.

	Total (*n* = 164)	g-CAP (*n* = 82)	r-CAP (*n* = 82)	*p* Value
CCI				>0.9999
0–1	114 (69.51%)	57 (69.51%)	57 (69.51%)	>0.9999
2–3	44 (26.83%)	22 (26.83%)	22 (26.83%)	>0.9999
4–5	6 (3.66%)	3 (3.66%)	3 (3.66%)	>0.9999
Gender				0.6337
Female	67 (40.85%)	35 (42.68%)	32 (39.02%)	
Male	97 (59.15%)	47 (57.32%)	50 (60.98%)	
Age (y/o)	60.96 ± 18.22	60.77 ± 18.70	61.15 ± 17.84	0.5398
Smoking history				0.1290
Yes	51 (31.09%)	21 (25.61%)	30 (36.59%)	
Never	113 (68.91%)	61 (74.39%)	52 (63.41%)	
Season				0.0265 *
Cold	96 (58.54%)	55 (67.07%)	41 (50.00%)	
Warm	68 (41.46%)	27 (32.93%)	41 (50.00%)	
COPD				0.0169 *
Yes	25 (15.25%)	7 (8.54%)	18 (21.95%)	
No	139 (84.75%)	75 (91.46%)	64 (78.05%)	
Diabetes Mellitus				0.7059
Yes	36 (21.96%)	17 (20.73%)	19 (23.17%)	
No	128 (78.04%)	65 (79.27%)	63 (76.83%)	
Hypertension				0.8738
Yes	67 (40.85%)	33 (40.24%)	34 (41.46%)	
No	97 (59.15%)	49 (59.76%)	48 (58.54%)	
Hyperlipemia				0.5021
Yes	52 (31.70%)	28 (34.15%)	24 (29.27%)	
No	112 (68.30%)	54 (65.85%)	58 (70.73%)	
COVID-19 history				0.1756
Yes	15 (9.15%)	10 (12.20%)	5 (6.10%)	
No	149 (90.85%)	72 (87.80%)	77 (93.90%)	
T_O-A_ (days)	30.66 ± 79.05	11.48 ± 15.77	49.85 ± 107.6	<0.0001 ****
T_O-A_ ≥ 7 days	117 (71.34%)	53 (64.63%)	64 (78.05%)	0.0836
T_O-A_ > 30 days	22 (13.42%)	3 (3.66%)	19 (23.17%)	0.0004 ***
Anti-microbial therapy before admission				0.0001 ***
Quinolones	141 (86.60%)	72 (87.80%)	69 (84.15%)	0.4999
Semisynthetic penicillin	22 (13.42%)	8 (9.76%)	14 (17.07%)	0.2516
Tetracycline	4 (2.44%)	2 (2.44%)	2 (2.44%)	>0.9999
Cephalosporin (I, II)	26 (15.85%)	3 (3.66%)	23 (28.05%)	<0.0001 ****
Macrolides	10 (6.10%)	4 (4.88%)	6 (7.32%)	0.5140
Neuraminidase inhibitor	6 (3.66%)	2 (2.44%)	4 (4.88%)	0.4055
Anti-SARS-CoV-2 medicine ^#^	6 (3.66%)	6 (7.32%)	0 (0.00%)	0.0126 *
Carbapenem	9 (5.49%)	0 (0.00%)	9 (10.98%)	0.0020 **
SCAP				<0.0001 ****
Yes	15 (9.15%)	0 (0.00%)	15 (18.29%)	
No	149 (90.85%)	82 (100.00%)	67 (81.71%)	
CURB-65				0.9911
0	76 (46.34%)	39 (47.56%)	37 (45.12%)	0.7541
1	58 (35.37%)	29 (35.37%)	29 (35.37%)	>0.9999
2	26 (15.85%)	13 (15.85%)	13 (15.85%)	>0.9999
3	3 (1.83%)	1 (1.22%)	2 (2.44%)	>0.9999
4	1 (0.61%)	0 (0.00%)	1 (1.22%)	>0.9999
PSI				0.1266
I	35 (21.34%)	22 (26.83%)	13 (15.85%)	0.0863
II	128 (78.05%)	60 (73.17%)	68 (82.93%)	0.1312
III	1 (0.61%)	0 (0.00%)	1 (1.22%)	>0.9999
Outcome				0.0011 **
Cured/Improved	154 (93.90%)	82 (100.00%)	72 (87.80%)	
Deteriorated/Death	10 (6.10%)	0 (0.00%)	10 (12.20%)	

CCI: Charlson Comorbidity Index; COPD: chronic obstructive pulmonary disease; COVID-19: coronavirus disease 2019; SARS-CoV-2: severe acute respiratory syndrome coronavirus 2; T_O-A_: time from onset to admission; SCAP: severe community-acquired pneumonia. ^#^ Anti-SARS-CoV-2 medicine: including Nematovir/Litonavir, Molnupiravir, Azvudine, Simnotrelvir/Ritonavir, and Atilotrelvir/Ritonavir. * *p* < 0.05, ** *p* < 0.01, *** *p* < 0.001, **** *p* < 0.0001.

**Table 2:** Peripheral lymphocyte subsets.

	g-CAP (*n* = 82)	r-CAP (*n* = 82)	*p* Value
WBC (×10^9^/L)	7.13 (5.94, 8.75)	7.57 (5.83, 10.63)	0.1610
Lym (×10^9^/L)	1.27 (0.94,1.78)	1.38 (1.01,1.70)	0.7958
Lym%WBC (%)	19.35 ± 7.85	19.11 ± 9.09	0.8621
B (/µL)	139.00 (76.25, 209.30)	147.00 (66.00, 219.30)	0.7258
B%Lym (%)	12.39 (7.42,17.74)	12.88 (7.32, 16.69)	0.3836
T (/µL)	779.00 (533.00, 1125.00)	918.00 (537.00, 1135.00)	0.3446
T% (%)	70.12 (62.89, 76.37)	72.34 (66.28, 76.95)	0.0828
CD4^+^ (/µL)	457.00 (298.30, 669.50)	546.50 (359.30, 751.50)	0.0397 *
CD4^+^%Lym (%)	41.50 ± 8.19	47.50 ± 8.53	<0.0001 ****
CD8^+^ (/µL)	257.50 (160.00, 412.50)	252.50 (164.00, 372.30)	0.5928
CD8^+^%Lym (%)	23.37 (17.84, 28.77)	20.80 (16.52, 26.44)	0.1196
CD4^+^/CD8^+^	1.75 (1.30, 2.53)	2.20 (1.70, 3.03)	0.0033 **
DNT (/µL)	0.00 (0.00, 8.25)	0.00 (0.00, 0.00)	0.6496
DNT%Lym (%)	0.00 (0.00, 1.27)	0.00 (0.00, 0.00)	0.2565
NK (/µL)	178.00 (99.75, 259.40)	164.50 (93.75, 243.30)	0.2249
NK%Lym (%)	14.32 (10.08, 21.89)	13.66 (8.58, 18.28)	0.1000
Mo (×10^9^/L)	0.45 (0.32, 0.61)	0.48 (0.35, 0.61)	0.6789
Mo%WBC (%)	6.15 (4.78, 87.60)	6.35 (5.18, 7.83)	0.7941

WBC: white blood cell; Lym: lymphocyte; DNT: double-negative T cell; NK: natural killer cell; Mo: monocyte. * *p* < 0.05, ** *p* < 0.01, **** *p* < 0.0001.

**Table 3:** Laboratory indicators.

	g-CAP (*n* = 82)	r-CAP (*n* = 82)	*p* Value
TC (mmol/L)	4.08 (3.62, 4.71)	4.05 (3.16, 4.74)	0.0428 *
TG (mmol/L)	1.25 (0.97, 1.95)	1.16 (0.79, 1.60)	0.0056 **
LDL-C (mmol/L)	2.65 ± 0.70	2.58 ± 0.91	0.5849
HDL-C (mmol/L)	0.93 (0.79, 1.14)	0.96 (0.77, 1.19)	0.9804
Na^+^ (mmol/L)	138.00 (135.80, 140.00)	139.00 (136.00, 141.00)	0.0217 *
K^+^ (mmol/L)	3.86 ± 0.34	3.87 ± 0.38	0.7771
Cl^−^ (mmol/L)	103.00 (101.00, 105.00)	105.00 (102.00, 107.00)	0.0740
UA (µmol/L)	290.50 (241.50, 355.50)	293.00 (220.50, 375.50)	0.7888
Fe (µmol/L)	7.95 (4.97, 11.88)	6.30 (3.70, 13.80)	0.9076
TCa^2+^ (mmol/L)	2.24 (2.16, 2.34)	2.20 (2.11, 2.34)	0.0848
CCa^2+^ (mmol/L)	2.27 (2.21, 2.31)	2.29 (2.21, 2.33)	0.3547
FCa^2+^ (mmol/L)	1.08 (1.05, 1.11)	1.10 (1.06, 1.13)	0.0337 *
Mg^2+^ (mmol/L)	0.86 ± 0.08	0.86 ± 0.09	0.9851
CRP (mg/L)	18.03 (4.85, 59.31)	41.06 (5.39, 106.2)	0.0334 *

TC: total cholesterol; TG: total triglyceride; LDL-C: low-density lipoprotein cholesterin; HDL-C: high-density lipoprotein cholesterin; Na^+^: sodium; K^+^: potassium; Cl^−^: chloride; UA: uric acid; Fe: serum iron; TCa^2+^: total calcium; CCa^2+^: calculated calcium; FCa^2+^: free calcium; Mg^2+^: magnesium; CRP: C-reactive protein. * *p* < 0.05, ** *p* < 0.01.

**Table 4:** Correlative factors based on the logistic regression analysis.

Variate	Univariate OR (95%CI)	*p* Value	Multivariate OR (95%CI)	*p* Value
CCI	1.000 (0.497, 2.011)	>0.9999	0.205 (0.031, 1.265)	0.0909
Gender	1.164 (0.624, 2.176)	0.6338	0.615 (0.137, 2.611)	0.5138
Age (y/o)	1.001 (0.984, 1.018)	0.894	0.986 (0.950, 1.022)	0.442
Smoking history	1.676 (0.862, 3.303)	0.1305	0.346 (0.057, 1.848)	0.2253
Season	2.037 (1.088, 3.863)	0.0274 *	5.341 (1.305, 27.110)	0.0281 *
COPD	3.013 (1.229, 8.172)	0.0207 *	62.280 (5.909, 1197.00)	0.0019 **
Diabetes mellitus	1.153 (0.549, 2.436)	0.7061	6.411 (1.022, 48.450)	0.0551
Hypertension	1.052 (0.564, 1.964)	0.8738	1.385 (0.267, 7.488)	0.6980
Hyperlipemia	0.798 (0.411, 1.542)	0.5024	3.092 (0.591, 20.2)	0.2022
COVID-19 history	0.468 (0.140, 1.382)	0.1836	0.276 (0.00998, 4.916)	0.4031
T_O-A_ (days)	1.036 (1.015, 1.063)	0.0029 **	1.037 (1.008, 1.077)	0.0354 *
WBC (×10^9^/L)	1.059 (0.959, 1.174)	0.2658	1.996 (1.027, 4.458)	0.0621
Lym (×10^9^/L)	0.865 (0.574, 1.176)	0.4044	0.080 (0.00069, 1.769)	0.2428
Lym%WBC (%)	0.997 (0.961, 1.034)	0.8564	1.525 (1.131, 2.195)	0.0122 *
B (/µL)	0.999 (0.996, 1.002)	0.5082	0.986 (0.969, 1.001)	0.0663
B%Lym (%)	0.979 (0.935, 1.024)	0.3555	1.146 (0.878, 1.535)	0.3250
T (/µL)	1.000 (0.9996, 1.001)	0.4121	0.9997 (0.981, 1.006)	0.9450
T% (%)	1.030 (0.994, 1.069)	0.1111	0.386 (0.202, 0.650)	0.0012 **
CD4^+^ (/µL)	1.001 (1.000, 1.002)	0.0288 *	1.002 (0.994, 1.023)	0.7028
CD4^+^%Lym (%)	1.093 (1.049, 1.143)	<0.0001 ****	3.044 (1.766, 6.016)	0.0003 ***
CD8^+^ (/µL)	1.000 (0.999, 1.001)	0.7572	1.002 (1.000, 1.027)	0.5727
CD8^+^%Lym (%)	0.966 (0.927, 1.004)	0.0834	2.621 (1.561, 4.852)	0.0006 ***
CD4^+^/CD8^+^	1.440 (1.095, 1.969)	0.0142 *	1.711 (0.659, 4.471)	0.2516
DNT (/µL)	1.001 (0.995, 1.007)	0.8467	0.978 (0.917, 1.033)	0.4465
DNT%Lym (%)	1.004 (0.942, 1.072)	0.9082	2.188 (1.071, 5.18)	0.0420 *
NK (/µL)	0.999 (0.996 1.001)	0.2628	0.999 (0.983, 1.016)	0.8681
NK%Lym (%)	1.000 (0.980, 1.021)	0.9892	1.036 (0.998, 1.17)	0.1270
Mo (/µL)	0.838 (0.385, 1.235)	0.4740	0.589 (0.193, 1.624)	0.3145
Mo%WBC (%)	0.983 (0.916, 1.03)	0.5233	0.929 (0.838, 1.02)	0.1364
TC (mmol/L)	0.788 (0.588, 1.034)	0.0962	0.0086 (0.0001, 0.2967)	0.0173 *
TG (mmol/L)	0.641 (0.426, 0.9025)	0.0188 *	0.752 (0.313, 1.655)	0.4887
LDL-C (mmol/L)	0.896 (0.609, 1.311)	0.572	178.500 (4.435, 17,773.00)	0.0131 *
HDL-C (mmol/L)	0.864 (0.312, 2.371)	0.776	9.349 (0.105, 1385.00)	0.3427
Na^+^ (mmol/L)	1.119 (1.022, 1.239)	0.0231 *	1.429 (1.084, 2.005)	0.0206 *
K^+^ (mmol/L)	1.136 (0.485, 2.682)	0.7684	0.430 (0.053, 3.052)	0.4084
Cl^-^ (mmol/L)	1.040 (0.988, 1.112)	0.1769	0.956 (0.832, 1.088)	0.4871
UA (µmol/L)	0.9997 (0.997, 1.002)	0.8134	1.007 (0.9998, 1.015)	0.0700
Fe (µmol/L)	1.005 (0.955, 1.057)	0.858	1.061 (0.919, 1.230)	0.415
TCa^2+^ (mmol/L)	0.355 (0.047, 2.229)	0.2871	0.059 (0.000022, 130.8)	0.4712
CCa^2+^ (mmol/L)	3.102 (0.122, 86.89)	0.4953	0.0189 (0.0000015, 181.7)	0.3970
FCa^2+^ (mmol/L)	2255.00 (4.308, 2,154,700)	0.0206 *	1,917,152 (25.37, 1,026,463,384,926)	0.0170 *
Mg^2+^ (mmol/L)	0.964 (0.022, 42.420)	0.9847	493.10 (0.084, 5,552,542)	0.1699
CRP (mg/L)	1.006 (1.001, 1.011)	0.0250 *	1.022 (1.004, 1.043)	0.0230 *

CCI: Charlson Comorbidity Index; COPD: chronic obstructive pulmonary disease; COVID-19: coronavirus disease 2019; T_O-A_: time from onset to admission; WBC: white blood cell; Lym: lymphocyte; DNT: double-negative T cell; NK: natural killer cell; Mo: monocyte; TC: total cholesterol; TG: total triglyceride; LDL-C: low-density lipoprotein cholesterin; HDL-C: high-density lipoprotein cholesterin; Na^+^: sodium; K^+^: potassium; Cl^−^: chloride; UA: uric acid; Fe: serum iron; TCa^2+^: total calcium; CCa^2+^: calculated calcium; FCa^2+^: free calcium; Mg^2+^: magnesium; CRP: C-reactive protein. * *p* < 0.05, ** *p* < 0.01, *** *p* < 0.001, **** *p* < 0.0001.

**Table 5:** Power of ROC curves for r-CAP.

Variate	Sensitivity	Specificity	AUC	*p* Value	Pseudo R Squared	Hosmer- Lemeshow
Season	50.00%	67.07%	0.5854	0.0591	0.0300	>0.9999
COPD	21.95%	91.46%	0.5671	0.1380	0.0348	>0.9999
T_O-A_	48.78%	89.02%	0.7320	<0.0001 ****	0.1241	0.0352 *
Lym%	53.66%	48.78%	0.5071	0.8759	0.0002	0.4904
T%Lym	56.10%	52.44%	0.5709	0.1171	0.0158	0.4493
CD4^+^ T%Lym	63.41%	62.20%	0.6881	<0.0001 ****	0.1153	0.4338
CD8^+^ T%Lym	64.63%	53.66%	0.5699	0.1222	0.0186	0.4364
DNT%Lym	79.27%	24.39%	0.5247	0.5851	8.009 × 10^−5^	0.0640
CRP	45.12%	74.39%	0.5773	0.0872	0.0326	0.6439
TC	52.44%	48.78%	0.5608	0.1791	0.0173	0.0406 *
LDL-C	52.44%	50.00%	0.5378	0.4035	0.0020	0.2429
Na^+^	64.63%	46.34%	0.5973	0.0314 *	0.0391	0.9454
FCa^2+^	58.54%	54.88%	0.6096	0.0154	0.0350	0.9655
Combined ^#^	75.61%	76.83%	0.8711	<0.0001 ****	0.4235	0.7385

COPD: chronic obstructive pulmonary disease; T_O-A_: time from onset to admission; Lym: lymphocyte; DNT: double-negative T cell; TC: total cholesterol; LDL-C: low-density lipoprotein cholesterin; Na^+^: sodium; FCa^2+^: free calcium; CRP: C-reactive protein. ^#^ The indicators of the Combined Curve include season; COPD history; time from onset to admission; percentages of T, CD4^+^ T, CD8^+^ T, and DNT cells; and levels of CRP, TC, LDL-C, Na^+^, and FCa^2+^. * *p* < 0.05, **** *p* < 0.0001.

**Table 6:** Pathogen ranking for r-CAP (bacteria and fungi).

Bacterium (Top 10)	Detection Rate (%)	Fungus (Top 10)	Detection Rate (%)
Klebsiella pneumoniae	14.6%	Candida albicans	13.4%
Streptococcus pneumoniae	12.2%	Aspergillus fumigatus	8.5%
Staphylococcus aureus	8.5%	Pneumocystis jirovecii	6.1%
MRSA	2.4%		
Haemophilus influenzae	6.1%	Candida glabrata	4.9%
Mycobacterium tuberculosis complex	6.1%	Other Aspergillus ^#^	4.9%
Pseudomonas aeruginosa	4.9%	Candida parapsilosis	3.7%
Stenotrophomonas maltophilia	3.7%	Irpex lacteus	2.4%
Acinetobacter baumannii	2.4%	Schizophyllum commune	2.4%
Escherichia coli	2.4%	Cryptococcus neoformans	2.4%
Chlamydia pneumoniae	2.4%		
Moraxella catarrhalis	2.4%		
Staphylococcus caprae	2.4%		
Enterococcus faecium	2.4%		
Corynebacterium striatum	2.4%		
Streptococcus intermedius	2.4%		
Nocardia ^##^	2.4%		
Nontuberculosis mycobacteria	2.4%		

MRSA: *methicillin-resistant Staphylococcus aureus*. ^#^ Other *Aspergilli* include *Aspergillus glaucus* (2.4%), *Aspergillus ruber* (1.2%), and *Aspergillus sydowii* (1.2%). ^##^ Nocardia include *Nocardia abscessus* (1.2%) and *Nocardia otitidiscaviarum* (1.2%).

**Table 7:** Pathogen ranking for r-CAP (viruses and all pathogens).

Virus (Top 10)	Detection Rate (%)	All Pathogens (Top 15)	Detection Rate (%)
Human gammaherpesvirus 4/ Epstein-Barr virus	9.8%	Klebsiella pneumoniae	14.6%
Human alphaherpesvirus 1/Herpes simplex virus type 1	7.3%	Candida albicans	13.4%
Human betaherpesvirus 5/ Human cytomegalovirus	7.3%	Streptococcus pneumoniae	12.2%
Severe acute respiratory syndroame coronavirus 2	4.9%	Human gammaherpesvirus 4/Epstein-Barr virus	9.8%
Human betaherpesvirus 7	3.7%	Staphylococcus aureus	8.5%
Human picobirnavirus	2.4%	Aspergillus fumigatus	8.5%
Influenza A virus	2.4%	Human alphaherpesvirus 1	7.3%
Human coronavirus 229E	2.4%	Human betaherpesvirus 5/Human cytomegalovirus	7.3%
Human orthopneumovirus	2.4%	Haemophilus influenzae	6.1%
Human mastadenovirus	2.4%	Mycobacterium tuberculosis complex	6.1%
		Pneumocystis jirovecii	6.1%
		Pseudomonas aeruginosa	4.9%
		Candida glabrata	4.9%
		Other Aspergillus	4.9%
		Severe acute respiratory syndroame coronavirus 2	4.9%

## Data Availability

The data presented in this study are available from the corresponding author on request due to privacy.

## Abbreviations

The following abbreviations are used in this manuscript:

AIDS	acquired immunodeficiency disease
AMR	antimicrobial resistance
BALF	bronchoalveolar lavage fluid
CAP	community-acquired pneumonia
CBC	complete cell count
CCa^2+^	calculated calcium
CCI	Charlson Comorbidity Index
CMV	cytomegalovirus
COPD	chronic obstructive pulmonary disease
COVID-19	coronavirus disease 2019
CRP	C-reactive protein
CTL	cytotoxic T lymphocyte
CTL	cytotoxic T lymphocyte
DALYs	disability-adjusted life-years
DMARDs	disease-modifying anti-rheumatic drugs
DNT cell	double-negative T cell
EBV	*Epstein–Barr virus*
FCa^2+^	free calcium
GBD	Global Burden of Diseases
g-CAP	general community-acquired pneumonia
HDL-C	high-density lipoprotein cholesterin
HIV	human immunodeficiency virus
HHV	human herpes virus
IHME	Institute for Health Metrics and Evaluation
LDL-C	low-density lipoprotein cholesterin
MRSA	methicillin-resistant Staphylococcus aureus
NGS	next-generation sequencing
NK cell	natural killer cell
r-CAP	refractory community-acquired pneumonia
ROC curve	receiver operating characteristic curve
SCAP	severe community-acquired pneumonia
TC	total cholesterol
TCa^2+^	total calcium
T_EM_ cell	effectory memory T cell
TG	total triglyceride
T_RM_ cell	tissue-resident effectory memory T cell
UA	uric acid
WBC	white blood cell
